# Human adipose-derived mesenchymal stem cells alleviate obliterative bronchiolitis in a murine model via IDO

**DOI:** 10.1186/s12931-017-0599-5

**Published:** 2017-06-15

**Authors:** Guoping Zheng, Guanguan Qiu, Menghua Ge, Jianping He, Lanfang Huang, Ping Chen, Wei Wang, Qi Xu, Yaoqin Hu, Qiang Shu, Jianguo Xu

**Affiliations:** 1 Shaoxing Second Hospital, 123 Yanan Road, Shaoxing, Zhejiang 312000 China; 2 The Children’s Hospital of Zhejiang University School of Medicine, 3333 Binsheng Road, Hangzhou, Zhejiang 310051 China; 3 The First Affiliated Hospital of Zhejiang University School of Medicine, 79 Qingchun Road, Hangzhou, Zhejiang 310003 China

**Keywords:** Mesenchymal stem cells, Adipose-derived stem cells, Obliterative bronchiolitis, Heterotopic tracheal transplant, indoleamine 2,3-dioxygenase

## Abstract

**Background:**

Long-term survival of lung transplantation is hindered by the development of obliterative bronchiolitis (OB). Adipose-derived stem cells (ASCs) were documented to have more potent immunosuppressive ability than mesenchymal stem cells (MSCs) from bone marrow and placenta. The goal of our study is to evaluate the effect of repeated administration of ASCs on OB and the involvement of indoleamine 2,3-dioxygenase (IDO) mediating the protective effect of ASCs in a heterotopic tracheal transplantation (HTT) model.

**Methods:**

For studies in vitro, ASCs were treated with interferon-γ (IFN-γ). For in vivo study, tracheas from BALB/c or C57BL/6 donors were transplanted into C57BL/6 recipients to create a HTT model. On days 0, 1, 3, 5, 8, 12, 15, 20 and 25 post-transplant, the allogeneic recipient mice were administered intravenously with phosphate buffered saline, 1 × 10^6^ human ASCs, or 1 × 10^6^ human ASCs plus 1-methyltryptophan (1-MT), an IDO inhibitor. On days 3, 7, 14 and 28, serum, trachea and spleen samples were harvested for analysis.

**Results:**

ASCs homed to heterotopic tracheal grafts after infusion. Multiple doses of ASCs significantly increased tracheal IDO levels in allografts. There were significant increases in graft and serum IFN-γ levels in allografts compared with isografts. IFN-γ elevated IDO expression and activity in ASCs in vitro. ASCs alleviated OB in allografts as evidenced by reduced epithelial loss, epithelial apoptosis, and intraluminal obstruction. The effects of ASCs on OB were blocked by 1-MT. 1-MT also blocked the alterations in pro and anti-inflammatory cytokines as well as CD3+ T cell infiltration induced by ASCs. ASCs induced not only splenic levels of CD4+CD25+Foxp3+ regulatory T cells (Treg) but also IL-10 and TGF-β-producing Treg. Furthermore, IDO inhibition abolished the changes of splenic Treg induced by ASCs. In addition, Treg reduction by cyclophosphamide treatment did not alter the effects of ASCs on tracheal IDO expression in allografts confirming Treg induction is downstream of IDO.

**Conclusions:**

Repeated doses of ASCs are capable of ameliorating OB. ASCs act at least in part via elevating IDO expression. ASCs promote the generation of Treg and suppress T cell infiltration via an IDO-dependent mechanism.

## Background

Lung transplantation offers the only hope for survival in many end-stage pulmonary diseases. According to the official International Society for Heart and Lung Transplantation (ISHLT) Registry Report of 2015, the unadjusted survival rates after adult lung transplantation were 80% at 1 year, 65% at 3 years, 54% at 5 years, and 31% at 10 years between January 1990 and June 2013 with a median survival of 5.7 years [1]. The main threat to long-term survival of lung transplantation is obliterative bronchiolitis (OB) along with its associated syndrome, bronchiolitis obliterans syndrome (BOS). BOS developed in 50% of primary adult lung transplant recipients within 5 years of transplantation and in 76% by 10 years post-transplant [2]. Median survival after diagnosis is between 3 and 5 years. Pathologically, OB is characterized by obliteration of the small airways with a relatively normal surrounding parenchyma. The mechanisms for OB are not fully unraveled. Current hypothesis is that recurrent damage of the epithelium induces chronic inflammation in the small airways and fibrotic repair response. During the process, the epithelial to mesenchymal transition occurs, resulting in generation of (myo-) fibroblasts and remodeling of the airway [3]. Recent treatment with alemtuzumab and basiliximab induction therapy received only limited success in lowering risk of developing OB and improving survival [4].

MSCs are multipotent and self-renewable cells in the bone marrow (BM-MSCs), adipose tissue (adipose-derived stem cells, ASCs), and in the connective tissues of most organs. In addition to their differentiation properties, MSCs possess broad immunoregulatory properties [5]. The immunoregulatory capacities have made them an outstanding candidate for conditions in which inflammation and immunopathologic reactions have a fundamental role [6]. The beneficial role of MSCs appears to derive from the release of immunoregulatory paracrine factors including nitric oxide (NO), indoleamine 2,3-dioxygenase (IDO), tumor necrosis factor-stimulated gene 6 (TSG-6), interleukin-10 (IL-10), and prostaglandin E2 (PGE2) among others. Beneficial results have been demonstrated in clinical trials for graft-versus-host diseases, autoimmune diseases such as Crohn’s disease, myocardial infarction, and osteoarticular diseases [7]. Our group has published the promising findings of ASCs for acute respiratory distress syndrome [8]. Therefore, MSCs might be a potential candidate for treating OB.

Although both ASCs and BM-MSCs share many similar characteristics such as paracrine factors and differentiation properties, several studies reported that ASCs are more effective suppressors of immune response by directly comparing MSCs from different tissue sources. Both BM-MSCs and ASCs displayed immunosuppressive properties in a dose–response manner in mixed lymphocyte reaction. ASCs are more potent at low doses compared to BM-MSCs [9]. ASCs are more potent suppressors of dendritic cells differentiation compared to BM-MSCs [10]. Furthermore, ASCs showed a stronger inhibitory effect to prevent CD4^+^ and CD8^+^ T cell activation and acquisition of lymphoblast characteristics than BM-MSCs and MSCs from umbilical cord matrix [11]. In addition, ASCs are an attractive source of MSCs for clinical application due to their ease of accessibility and abundance. The yield of ASCs is 500-fold greater than BM-MSCs when isolated from an equivalent amount of tissue [12].

IDO is an intracellular enzyme that degrades tryptophan into kynurenine. Both depletion of tryptophan and accumulation of kynurenine are able to suppress T-cell proliferation [13]. IDO is one of the key immunoregulators secreted by MSCs, tumors and during pregnancy. During pregnancy, IDO is expressed in trophoblasts surrounding the embryo and protect the embryo from T cell mediated rejection. Inhibition of IDO activity during murine pregnancy results in rejection of embryo by the maternal immune system [14]. Expression of IDO by tumor cells in mice is accompanied by reduced accumulation of specific T cells at the tumor site. Treatment of the mice with an inhibitor of IDO results in tumor rejection [15]. MSCs have been shown to inhibit T cell activation and proliferation via IDO-mediated mechanisms [16]. IDO plays a role in the effects of MSCs in hind limb ischemia-reperfusion injury [17]. Moreover, IDO is associated with regulatory T cell (Treg) generation and kidney allograft tolerance induced by MSCs [18].

Several studies, including a publication from our group [19], have demonstrated the benefits of single dose of MSCs from bone marrow and placenta in alleviating OB of animal models [20–22]. The benefits appear derive from generation of IL-10 and Foxp3+ Treg [19, 22]. Since OB is a chronic disease, the present study is to determine the effects of multiple doses of ASCs on the pathogenesis of OB in a heterotopic tracheal transplantation (HTT) model. This study is also to investigate whether ASCs alleviate OB via an IDO-mediated mechanism. Furthermore, the study aims to examine the involvement of IDO in infiltration of CD3+ T cells and the generation of Treg.

## Methods

### Culture and characterization of ASCs

Lack of IDO activity was reported for murine MSCs [23]. Therefore, human ASCs were selected for the study. Normal human ASCs were purchased from ATCC (Cat # PCS-500-011, LOT 59753760, passage 2, Manassas, VA) and characterized by our group before [8]. ASCs from passages 6–7 were used for the study. Cells were maintained in media containing Dulbecco’s Modified Eagle’s Medium (DMEM)-low glucose supplemented with penicillin, streptomycin, and 2% fetal bovine serum (Thermo Fisher Scientific, Waltham, MA) plus EGF and FGF (R&D Systems, Minneapolis, MN). Cultures were incubated at 37 °C in a humidified chamber containing 5% CO2. The cells were treated with trypsin/EDTA and replated at a density of 4000 cells/cm^2^ when the cultures were close to confluence (>80%). The phenotype of expanded ASCs was characterized using flow cytometry. Cell surface expression of the following markers was analyzed: CD73, CD90, CD105, CD34, CD45, and CD4 (BD Biosciences, Franklin Lakes, New Jersey). To test the osteogenic differentiation of the expanded ASCs, cells were cultured with osteogenic medium containing 10% FBS, 0.2 mM L-ascorbic acid 2-phosphate and 0.01 M β-glycerophosphate in DMEM. The cultures were stained for alkaline phosphatase (ALP) activity after 2–3 weeks. To examine the adipogenic differentiation, ASCs were cultured in adipogenic medium composed of DMEM and 10% FBS supplemented with 10 μg/ml insulin, 100 nM dexamethasone, 250 μM isobutylmethylxanthine, and 200 μM indomethacin. The cultures were stained with Oil Red O after 2–3 weeks.

### IDO assays in vitro

ASCs were seeded in a six-well plate at 6000 cells/cm^2^ on day 0. On day 1, ASCs were treated with or without different doses of human IFN-γ (10–100 ng/ml) (Peprotech, London, UK) for 24 h. Cell samples were harvested for determination of IDO protein levels via Western blot analysis and IDO enzymatic activity. IDO enzymatic activity was assayed by the colorimetric method with minor modifications. Briefly, 2 × 10^6^ IFN-γ-treated ASCs were lysed by freezing and thawing. The lysate (250 μl) was harvested by centrifugation, and an equal amount of 2 × IDO buffer [100 mM phosphate-buffered saline (PBS), pH 6.5, with 40 mM ascorbate, 20 μM methylene blue, 200 μg/ml catalase, and 800 mM l-tryptophan (Sigma–Aldrich, St. Louis, MO)] was added. After a 30 min incubation at 37 °C to permit IDO to convert tryptophan to kynurenine, 100 μl of 30% trichloroacetic acid was added to stop the reaction. The reaction mixture was then incubated for 30 min at 52 °C and centrifuged. The supernatant was mixed with an equal amount of Ehrlich’s reagent. The absorbance at 490 nm was measured in a spectrophotometer after 10 min of incubation at room temperature. One unit of IDO activity is defined as the amount of enzyme required to produce 1 nmol of kynurenine per hr. Protein concentration of the cell extracts was measured by BCA protein assay kit (Thermo Fisher Scientific).

### Animal maintenance

Experiments were conducted using 6 to 8-week-old C57BL/6 and BALB/c mice (Shanghai Laboratory Animal Center, Shanghai, China). All mice were housed in the Zhejiang University Laboratory Animal Center. All animals had free access to food and water. Animal experiment protocols were approved by the review committee from Zhejiang University School of Medicine and were in compliance with institutional animal care and use committee.

### HTT model

Tracheas were transplanted as previously described from our group [19, 24]. Donor tracheal segments were heterotopically transplanted into C57BL/6 recipients. For allografts, BALB/c tracheas were transplanted into C57BL/6 recipients. Both recipient and donor were C57BL/6 mice for isograft controls. Briefly, Tracheas were resected from freshly euthanized donor mice. The tracheas were immediately placed in ice-cold PBS with penicillin G sodium (100 U/ml) and streptomycin sulfate (100 μg/ml) (Life Technologies). C57BL/6 recipient mice were anesthetized with ketamine/xylazine (100 and 2 mg/kg intraperitoneally; Phoenix Pharmaceuticals, St. Joseph, MO). A 0.5 cm × 0.5 cm cross-shaped incision was made through the skin on the back of the recipient mouse. Four subcutaneous pockets were formed by blunt dissection. One tracheal graft was placed heterotopically into each subcutaneous pocket and incisions closed with suture. No immunosuppressive agents were given to any graft recipient.

### Animal treatment and examining homing of ASCs to allografts

Allogeneic C57BL/6 recipient mice were randomized to one of three experimental groups: 1) PBS control; 2) ASCs; or 3) ASCs + 1-methyltryptophan (1-MT, Sigma-Aldrich, St. Louis, MO). ASCs were washed with warm PBS and resuspended at a concentration of 1 × 10^6^ cells per 0.2 ml of PBS. ASCs (1 × 10^6^ cells) or PBS (0.2 ml) were injected via the tail veins of the mice. 1-MT was given by oral gavage (10 mg/0.4 ml/mice). Both ASCs and 1-MT were administered immediately after the transplant and on days 1, 3, 5, 8, 12, 15, 20 and 25 post-transplant. On days 3, 7, 14 and 28, serum, trachea and spleen samples were harvested for analysis. For Treg depletion experiment, ASCs (1 × 10^6^ cells/0.2 ml) or PBS (0.2 ml) were delivered to the allograft mice via tail veins on days 0, 1, 3, and 5 post-transplant. Mice were treated with or without 150 mg/Kg cyclophosphamide intraperitoneally on days 0, 1, 3, and 5 post-transplant. Spleen and tracheal grafts were harvested on day 7 for examination. To study the homing of ASCs to transplanted allografts, cultured ASCs were suspended with PBS at a density of 1 × 10^6^/ml and mixed with Vybrant™ DiD cell-labeling solution (5 μl/ml, Thermo Fisher Scientific) or PBS control. Cells were then incubated at 37 °C for 15 min in the dark, washed with PBS, resuspended at concentration of 1 × 10^6^ cells per 0.2 ml PBS, and injected via tail veins into allograft mice immediately after HTT. Mice were sacrificed 24 h after ASCs infusion to harvest the grafts. The tracheal grafts were diced with a clean blade and digested with collagenase A (Roche) and DNase I (Sigma-Aldrich) at 37 °C for 60 min to generate single cells. The cells were filtered through a 40-μm cell strainer (BD Bioscience, Franklins Lakes, NJ), centrifuged for 5 min at 500 *g*, and detected via flow cytometry for DiD+ cells.

### Determination of tracheal epithelial loss and apoptosis as well as luminal occlusion

Tracheal grafts were fixed in formalin, embedded, cross-sectioned, and stained with hematoxylin and eosin (H&E). Tracheal sections were photographed at 100 X magnification. The epithelial loss, epithelial apoptosis, and luminal occlusion were assessed by an independent, blinded reader. The luminal occlusion was measured using ImageJ software. The percentage of airway obstruction was calculated using the following formula: the area of the obliterated lumen divided by the total area of lumen × 100%. Percent of epithelial loss was calculated using the formula: (1- length of intact epithelialization divided by lumenal circumference) × 100%. For apoptosis analysis, the transverse sections of the grafts were subjected to terminal deoxynucleotidyl transferase dUTP nick end labeling (TUNEL) using the in situ Cell Death Detection Kit (Roche Applied Science, Mannheim, Germany) according to the manufacturer’s instruction. TUNEL positive cells were identified in the tracheal submucosa and airway epithelium with dark brown staining. Five to 10 grafts were assayed in each group.

### Serum cytokine ELISA

Serums were harvested as described above and stored at −80 °C. IFN-γ, IL-10, IL-6, and tumor necrosis factor-α (TNF-α) levels in the serum were determined via commercial ELISA kits (R&D Systems) according to the manufacturer’s instructions.

### Western blot

Harvested tracheas were homogenized in protein extract solution containing 0.1% Triton X-100, 100 mM NaCl, 10 mM HEPES (pH 7.9), 1 mM ethylene-diamine tetraacetic acid and 0.5 mM phenylmethanesulfonylfluoride (Sigma-Aldrich USA, St. Louis, MO) on ice, and centrifuged at 13,000 *g* for 10 min at 4 °C. The protein concentration was determined using a BCA protein assay kit. Protein extracts (20 μg) were separated on sodium dodecyl sulfate-polyacrylamide gel electrophoresis (SDS-PAGE) and transferred to polyvinylidenefluoride membranes (EMD Millipore, Billerica, MA). Membranes were blocked with a “sealed liquid” (5% non-fat dry milk in 1 × TBS) for 1 h at room temperature. Then, membranes were incubated with an IDO antibody (Catalog, MAB5412, EMD Millipore) for at 4 °C overnight. The blots were then washed three times with TBST buffer (150 mM NaCl, 10 mM Tris–HCl, pH 7.4, 0.1% Tween 20) and incubated for 1 h at room temperature with a horseradish peroxidase-conjugated secondary antibody (Catalog, 70-GAM0072, MultiSciences). Finally, the blots were washed three more times with TBST and visualized via enzyme-linked chemiluminescence using the EZ-ECL kit (Biological Industries, Kibbutz Beit-Haemek, Israel). Expression of each band was normalized to its corresponding glyceraldehyde-3-phosphate dehydrogenase (GAPDH) band.

### Quantitative real-time RT-PCR (qRT-PCR) analysis

Total RNA from tracheal grafts was isolated using Trizol (Thermo Fisher Scientific). After quantification of nucleic acids by spectrophotometry (Nanodrop, Thermo Fisher Scientific), 2 μg of the total RNA was reverse transcribed using PrimeScript RT Reagent Kit (Takara). cDNA products were amplified on a ABI 7500 Fast Real-Time PCR System in 20 μL of reaction mixture containing the SYBR GreenER qPCR Super-Mix Universal (Takara) and 10 μM of forward and reverse primers: IDO forward: GAGTAGACAGCAATGGCA; IDO reverse: AGTGGATGTGGTAGAGCA; β-actin forward: CTACAATGAGCTGCGTGTG; β-actin reverse: GCGTGAGGGAGAGCATAG (95 °C for 15 s, 55 °C for 1 min, and 65 °C for 1 min, 35 cycles). Results are expressed relative to the reference gene β-actin.

### Immunohistochemistry and histology

To determine the T cell infiltration, tracheal sections were stained with an rabbit anti mouse CD3 antibody (Catalog, ab16669, Abcam). After incubation with biotin-conjugated secondary antibody and an avidin-biotin complex, immunoreactivity was detected by incubating the sections with DAB to produce a brown color (Vector Laboratories, Burlingame, CA). Nuclei were detected with hematoxylin counterstaining. Negative controls were performed by omitting the primary antibody. The number of positively stained T cells were counted at 10 randomly selected high-power magnification fields (×400) in three histological sections per mouse from a total of four mice by an independent examiner.

### Flow cytometry analysis of splenic total, IL-10+, and TGF-β+ CD4+CD25+Foxp3+ Treg

Spleen were obtained from the mice and processed to achieve single cell suspensions by pressing the spleen through a 40-μm cell strainer in PBS. The red blood cells in splenic suspensions were lysed with 0.75% NH_4_Cl and Tris buffer (0.02%) (pH = 7.4) for 5 min. For surface staining, splenocytes were first incubated with PE-Cy5-conjugated anti–mouse CD4 and APC-conjugated anti–mouse CD25 (ebioscience) for 30 min at 4 °C. Cells were further fixed and permeabilized with Cytofix/Cytoperm solution (Catalog, 85-00-5523-00, ebioscience). Then, cells were stained with PE-conjugated anti-mouse Foxp3 (ebioscience), Brilliant Violet 421™-conjugated anti-mouse IL-10 (Biolegend), and Brilliant Violet 421™-conjugated anti-mouse TGF-β (Biolegend) for 30 min at 4 °C. Cells were analyzed using a BD FACSort flow cytometer (BD Biosciences) by gating CD4+ cells and examining the percentage of CD25+Foxp3+ Treg as well as IL10+/TGF-β + Treg. A minimum of 30,000 events was acquired for each sample. Isotype-matched control antibodies were used to determine the cut-off between negative and positive populations. Data were analyzed using FlowJo software (Tree Star Inc., Ashland, OR).

### Statistical methods

Data analysis was conducted using the Prism 5 software (GraphPad Software, Inc. CA, Prism 5.01). All values are presented as the means ± standard error of the mean (SEM). For statistical comparison among groups, one-way analysis of variance (ANOVA) was used. Results with a *p* < 0.05 were considered to be statistically significant.

## Results

### Characterization of the expanded ASCs

**Fig. 1:**
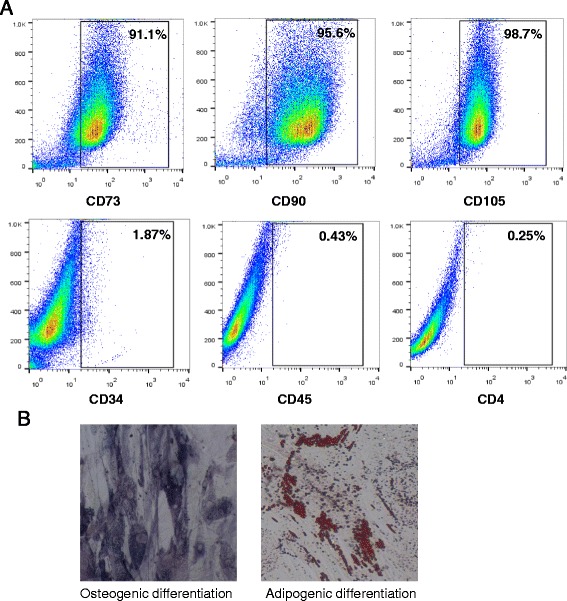
Phenotypic characterization and differentiation potential of ASCs. **a** Phenotypes for human ASCs were analyzed for surface markers via flow cytometry after expansion. ASCs positively express CD73, CD90, and CD105, but not CD34, CD45, and CD4. **b** For osteogenic differentiation, cells were stained for ALP activity. For adipogenic differentiation, cells were stained with Oil Red O. Magnification, 200×

ASCs were cultured and expanded by plastic adherence. To ensure the presence of ASCs and the absence of CD4+CD25+Foxp3+ Treg in the culture, surface protein expression at the end of expansion was characterized for the expression of ASCs markers. These cells were strongly positive for CD73 (91.1%), CD90 (95.6%) and CD105 (98.7%), but negative for CD34 (1.87%), CD45 (0.43%), and CD4 (0.25%) (Fig. 1a). The expanded ASCs possessed the abilities of osteogenesis as shown in alkaline phosphatase staining and adipogenesis as assayed by Oil Red O staining (Fig. 1b).

### ASCs engraft in tracheal allografts in HTT model and elevate IDO expression

**Fig. 2:**
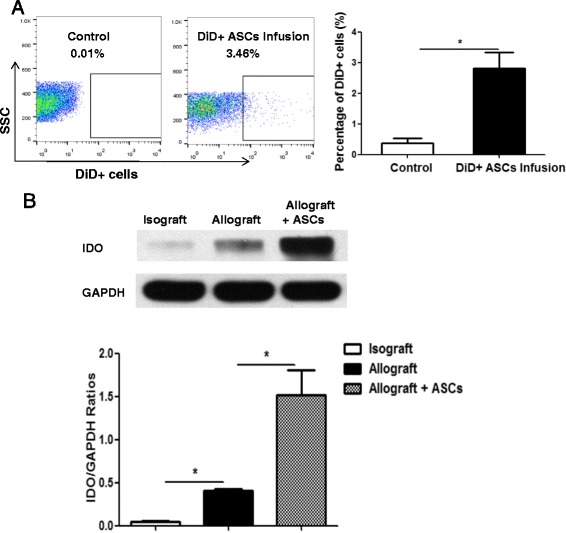
ASCs infusion on engraftment and IDO expression. C57BL/6 mice were transplanted heterotopically with tracheas from C57BL/6 (isograft) or BALB/c (allograft). **a** 1 × 10^6^ ASCs labeled with Vybrant™ DiD or PBS (0.2 ml) were administered to the recipient mice via tail veins immediately after allograft transplantation. Grafts were harvested and analyzed for the presence of labeled ASCs 24 h later via flow cytometry. **b** In the allograft group, 1 × 10^6^ human ASCs or PBS were administered to the recipient mice via tail veins on days 0, 1, 3, and 5 post-transplant. Tracheal grafts were harvested on day 7 post-transplant. Expression of IDO protein levels in the grafts were determined via Western blot analysis. The levels of GAPDH expression were assessed as loading controls. All data are expressed as mean ± SEM; *n* = 5 per group. *, *p* < 0.05

A HTT model was employed for the study. In allograft, donor tracheal segments from BALB/c were transplanted heterotopically into C57BL/6 recipients. To examine whether ASCs engraft to tracheal allografts, Vybrant™ DiD-labeled ASCs were infused to the allograft mice via tail veins. Twenty-four hours later, single cells isolated from tracheal allografts were examined via flow cytometry. The results showed that approximately 3% of cells in the allografts were positive for fluorescence demonstrating that ASCs home to the site of injury and inflammation (Fig. 2a).

It has been reported that while elevated IDO was present in allogeneic corneas at rejection, over-expression of IDO in donor cornea was found to significantly extend survival of allografts [25]. To determine the effects of tracheal transplantation and ASCs infusion on IDO levels, donor tracheal segments from BALB/c and C57BL/6 were transplanted heterotopically into C57BL/6 recipients. Human ASCs (1 × 10^6^/mouse) were administered to allograft mice on days 0, 1, 3, and 5 post-transplant. IDO protein levels in the tracheal grafts were determined via Western blot analysis on day 7. Low levels of IDO protein were detected in the isograft group (Fig. 2b). IDO levels were significantly increased in the allografts compared with the isografts (*p* < 0.05). Treatment with multiple doses of ASCs in the allograft mice further elevated the IDO expression compared with the allografts alone (*p* < 0.05) (Fig. 2b).

### IFN-γ elevates IDO expression in ASCs

**Fig. 3:**
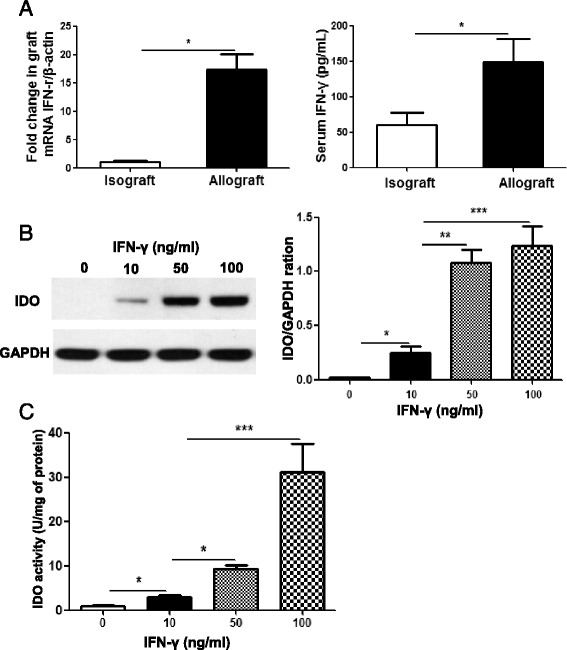
Mechanisms of increased IDO expression. **a** Grafts and serum samples were collected on day 7 post-transplant from the isograft and allograft mice. IFN-γ levels in the grafts were determined via RT-PCR. Serum IDO levels were quantitated via ELISA. **b** ASCs were stimulated with indicated concentrations of IFN-γ (10, 50 and 100 ng/ml) for 24 h. Protein lysates were extracted from the cells and the expression levels of IDO quantitated by Western blot analysis. GAPDH was used as loading control. **c** ASCs were lysed after IFN-γ (10, 50 and 100 ng/ml) stimulation for 24 h. Cell lysates were assayed for IDO enzymatic activity via colorimetric assay with Ehrlich’s reagent. All data are expressed as mean ± SEM; *n* = 4 per group. *, *p* < 0.05; **, *p* < 0.01 and ***, *p* < 0.001

IFN-γ is a potent inducer for IDO [26]. To study the involvement of IFN-γ in the increased expression of IDO in allografts, graft IFN-γ mRNA and serum IFN-γ protein levels were determined 7 days after transplantation in isograft and allograft groups treated as the above. The data revealed that both graft and serum IFN-γ levels were significantly elevated in allografts compared with isografts (Fig. 3a, *p* < 0.05). To study the direct effects of IFN-γ on IDO expression in ASCs, human ASCs were exposed to different concentration of IFN-γ for 24 h. Unstimulated ASCs did not constitutively express IDO protein. Treatment of ASCs with IFN-γ induced IDO protein expression (Fig. 3b) as well as enzyme activity (Fig. 3c) in a dose-dependent manner.

### ASCs attenuate OB via IDO dependent mechanisms

**Fig. 4:**
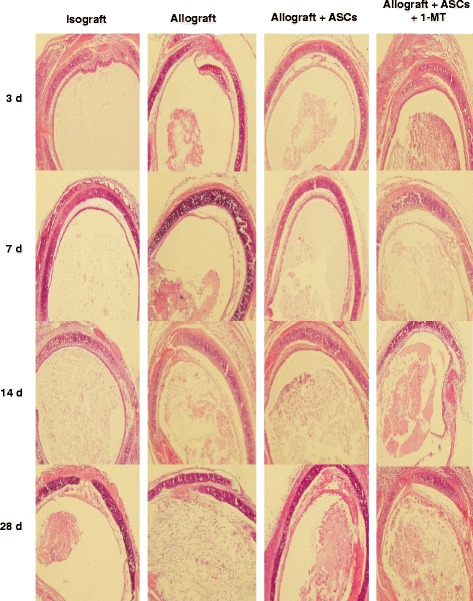
Effect of IDO inhibitor 1-MT and ASCs on tracheal allografts. C57BL/6 mice were transplanted heterotopically with tracheas from C57BL/6 (isograft) or BALB/c (allograft). In the allograft group, PBS (0.2 ml, IV), ASCs (1 × 10^6^ cells/0.2 ml, IV), or ASCs + 1-MT (10 mg, oral gavage) were administered to recipient mice on days 0, 1, 3, 5, 8, 12, 15, 20 and 25 post-transplant. Tracheal grafts were harvested on days 3, 7, 14, and 28 post-transplant. Histopathologic sections of grafts were stained with H&E. All images were 100 X magnification

**Fig. 5:**
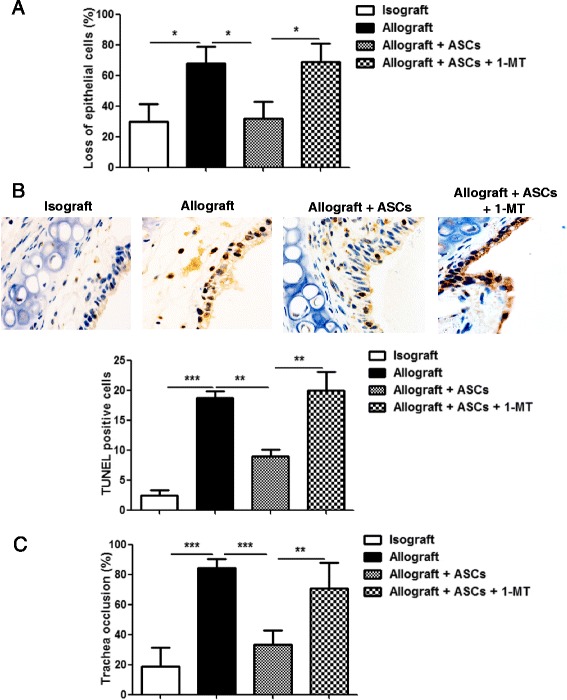
Effects of IDO inhibitor on epithelial integrity, epithelial apoptosis, and tracheal occlusion in allograft mice with ASCs treatment. Tracheal grafts described above were harvested on days 14 and 28 post-transplant and fixed with paraformaldehyde. **a** Tracheal sections were stained with H&E. Percentages of epithelial loss was determined on day 14 post-transplant. **b** The transverse sections of tracheal grafts on day 14 were examined with TUNEL assay. Cells with dark brown staining nucleus were apoptotic cells. The graph represented average number of TUNEL positive cells in a section. **c** Percentages of tracheal occlusion were determined on day 28 post-transplant. All data are expressed as mean ± SEM; *n* = 5 per group. *, *p* < 0.05; **, *p* < 0.01 and ***, *p* < 0.001

OB is a disease of chronic allograft rejection. To determine whether ASCs alleviate OB in HTT model via IDO dependent mechanisms, recipient mice were divided into four groups: isograft, allograft, allograft + ASCs, and allograft + ASCs + 1-MT (an IDO inhibitor). Human ASCs (1 × 10^6^/mouse) were administered to allograft mice on days 0, 1, 3, 5, 8, 12, 15, 20 and 25 post-transplant with or without IDO inhibitor 1-MT. Two allograft recipient mice died during the surgical operation but no mice died during 28 day experimental period after surgery. Tracheal grafts were harvested on days 3, 7, 14, and 28 post-transplant, fixed, embedded, and sectioned for staining with H&E. There were dramatic differences in inflammation, epithelial disruption and luminal space observed in the allograft tracheas compared with the isografts. These changes were alleviated in animals received ASCs. Treatment with 1-MT disrupted the effects of ASCs (Fig. 4). Treatment with ASCs significantly reduced intraluminal epithelial loss on day 14 post-transplant compared with the allograft group (28.0% ± 11.9% vs 66.9% ± 9.8%, *p* < 0.05) (Fig. 5a). ASCs decreased epithelial apoptosis on day 14 post-transplant as evidenced by significant less TUNEL positive cells in epithelium compared to allograft alone (*p* < 0.01) (Fig. 5b). ASCs also significantly decreased luminal occlusion on day 28 after transplantation compared with the allograft group (32.9% ± 8.6% vs 82.7% ± 12.6%, *p* < 0.001) (Fig. 5c). Treatment with 1-MT abolished the effect of ASCs on epithelial loss, epithelial apoptosis, and luminal occlusion (Fig. 5a, b, and c). These results indicate that IDO facilitates the protective role of ASCs.

### ASCs-induced modulation of cytokine expression is mediated by IDO

**Fig. 6:**
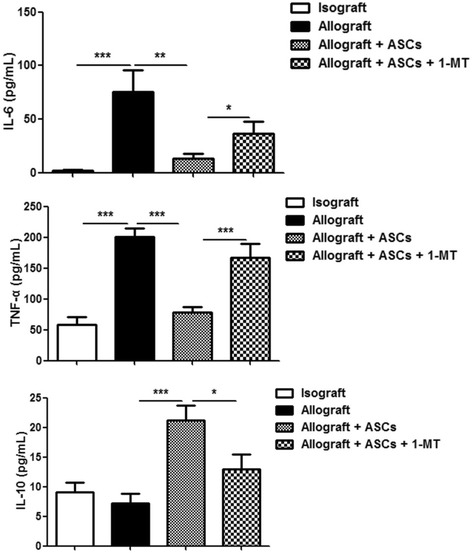
Effects of IDO inhibitor on cytokine levels in the serum of recipient mice. Serum samples were harvested on day 14 post-transplant from the four groups of recipient mice: isograft, allograft, allograft + ASCs, and allograft + ASCs + 1-MT. Cytokine levels in recipient mice were determined via ELISA. All data are expressed as mean ± SEM; *n* = 5 per group. *, *p* < 0.05; **, *p* < 0.01 and ***, *p* < 0.001

To examine the mechanisms of the modulatory effect of ASCs, serum cytokine levels were determined 14 days after transplantation from the 4 groups of mice (isograft, allograft, allograft + ASCs, and allograft + ASCs + 1-MT). The results showed that allografts had significantly higher proinflammatory IL-6 and TNF-α levels than isografts. Treatment with ASCs significantly decreased IL-6 and TNF-α levels in the allograft group. ASCs treatment also increased anti-inflammatory IL-10 level in the allografts (*p* < 0.001). Furthermore, the effects of ASCs on IL-6, TNF-α, and IL-10 levels were blocked by IDO inhibitor 1-MT (Fig. 6).

### IDO plays a role in the effects of ASCs on infiltration of CD3+ T cells

**Fig. 7:**
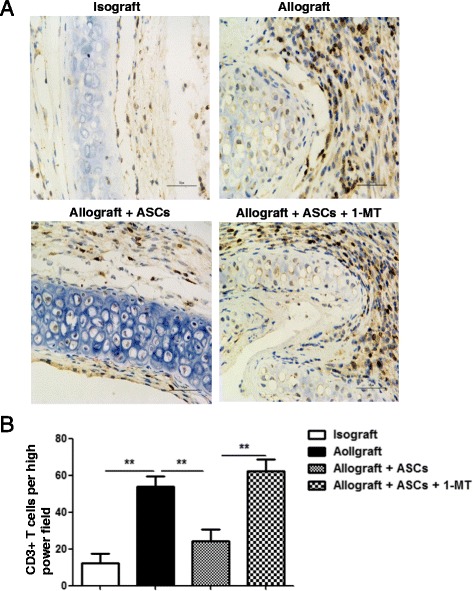
Effect of IDO inhibitor on CD3+ T cell infiltration in allografts with ASCs treatment. **a** Tissue sections from tracheal grafts on day 14 were immunostained with CD3 antibody and counterstained lightly with hematoxylin for viewing negatively stained cells. Cells stained with dark brown indicate CD3+ T-cell infiltration. **b** CD3+ T cells were quantitated from random fields under high magnification (400 ×). All data are expressed as mean ± SEM; *n* = 4 per group. **, *p* < 0.01

To determine the possible mechanism by which ASCs ameliorate OB, immunohistochemical analysis was performed to determine the levels of CD3+ T cells in the grafts on day 14 (Fig. 7a). Allografts had significant higher number of invaded CD3+ T cells than isografts (*p* < 0.01). Treatment with ASCs in allografts significantly reduced the infiltration of CD3+ T cells compared with allografts received PBS (*p* < 0.01). The effect of ASCs on CD3+ T cell infiltration was abolished by IDO inhibitor 1-MT (*p* < 0.01) (Fig. 7b).

### ASCs expand splenic CD4+CD25+Foxp3+ Treg via IDO

**Fig. 8:**
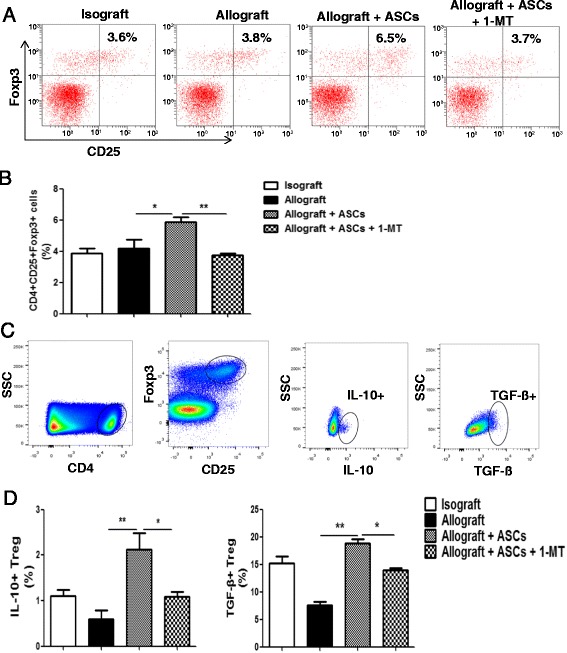
IDO inhibitor on the frequency of Treg and IL-10+/TGF-β+ Treg in recipient spleens. C57BL/6 mice were transplanted heterotopically with tracheas from BALB/c or C57BL/6 mice. Spleens were harvested on day 14 post-transplant. **a** Splenic cells were gated with CD4 and analyzed for CD25+ and Foxp3+ phenotype expression using flow cytometry. Representative dot plots were shown for the four experimental groups. **b** Frequency of splenic CD4+CD25+Foxp3+ Treg in each treatment group was summarized graphically. **c** Representative flow cytometry plots of the IL-10+ and TGF-β+ Treg in recipient spleens were presented. Spleen cells were initially gated by CD4 followed by CD25 and Foxp3. The CD4+CD25+Foxp3+ Treg were further gated for IL-10 and TGF-β. **d** The bar graphs summarized the percentages of IL-10+ and TGF-β+ Treg in isograft, allograft, allograft + ASCs, and allograft + ASCs + 1-MT. All data are expressed as mean ± SEM; *n* = 5 per group. *, *p* < 0.05 and **, *p* < 0.01

To test the hypothesis that ASCs induce CD4+CD25+Foxp3+ Treg via IDO, spleen tissues from the 4 different groups as outlined above were analyzed for CD4+CD25+Foxp3+ Treg expression by flow cytometry (Fig. 8a). The frequency of Treg was calculated as the percentage of CD25+Foxp3+ cells in the CD4+ population. Spleen cells obtained on day 14 post-transplant showed no difference in the frequency of Treg between allograft and isograft groups. Allograft mice received ASCs had a significant higher percentage of splenic Treg frequency than the allograft group received PBS (*p* < 0.05). Furthermore, the elevation of Treg induced by ASCs were abrogated by IDO inhibitor 1-MT (Fig. 8b, *p* < 0.01). Treg can exert their function via production of cytokines like IL-10 and TGF-β. To determine the effects of ASCs on cytokine production in splenic Treg from the above 4 groups, intracellular secretion of IL-10 and TGF-β was examined via flow cytometry (Fig. 8c). Allograft mice received ASCs had higher intracellular production of IL-10 and TGF-β than the allograft group (Fig. 8d, *p* < 0.01). Furthermore, 1-MT treatment diminished the elevation of IL-10 and TGF-β producing Treg (Fig. 8d, *p* < 0.05).

### Effect of ASCs on IDO expression is Treg independent

**Fig. 9:**
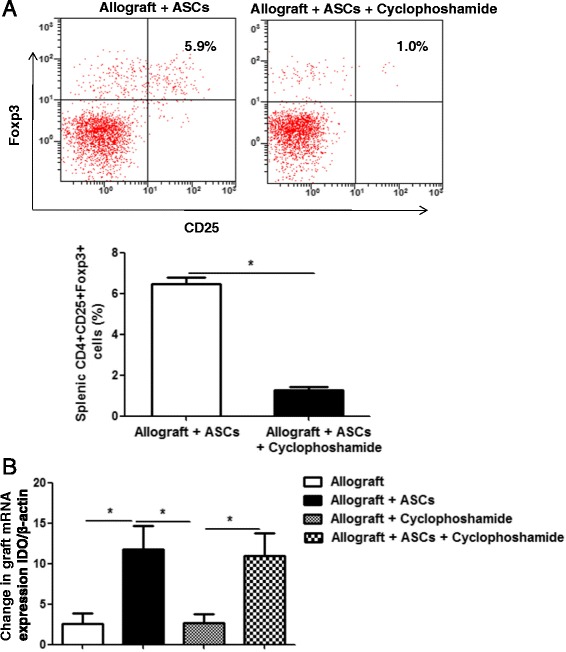
Effect of Treg depletion on IDO expression in tracheal allografts. C57BL/6 mice were transplanted heterotopically with tracheas from BALB/c. Human ASCs (1 × 10^6^/0.2 ml) or PBS were administered to the recipient mice via tail veins on days 0, 1, 3, and 5 post-transplant. Also, mice were treated with or without 150 mg/Kg cyclophosphamide intraperitoneally on days 0, 1, 3, and 5 post-transplant to deplete Treg. **a** Percentages of splenic CD4+CD25+Foxp3+ Treg on day 7 were shown in mice with and without Treg depletion. **b** Tracheal grafts were harvested on day 7 post-transplant. IDO expression in the grafts were determined via quantitative RT-PCR. β-actin mRNA was served as internal control. All data are expressed as mean ± SEM; *n* = 5 per group. *, *p* < 0.05

Many reports document that IDO can active Treg [27], while others suggest that Treg increase IDO levels [28]. IDO inhibition experiment in Fig. 8b indicates that IDO is upstream of Treg induction. To determine whether ASCs elevate IDO levels directly or indirectly via inducing Treg in vivo, allograft mice were treated with cyclophosphamide to deplete Treg. Cyclophosphamide treatment significantly reduced the splenic CD4+CD25+Foxp3+ Treg as determined in flow cytometry (Fig. 9a, *p* < 0.05). Nevertheless, ASCs-induced IDO expression in the tracheal allografts was unaffected after cyclophosphamide treatment (Fig. 9b). These results demonstrate that effect of ASCs on IDO expression is Treg independent.

## Discussion

Many studies have been conducted to determine the application of MSCs in organ transplantation due to their immunoregulatory properties. The beneficial effects of MSCs from bone marrow and placenta have been reported in animal models of OB by our group and other investigators [19–22]. Although ASCs were reported to be more potent in immunomodulation than bone marrow and placenta MSCs [9–11], the effects and mechanism of ASCs on OB are not understood. In the present study, we reported that human ASCs were able to engraft to tracheal allografts, elevate IDO expression, and alleviate OB. The expression of IDO was induced by IFN-γ in vitro. IDO inhibitor blocked the effects of ASCs on OB development, cytokine expression in the serum, and T cell infiltration in the allografts. In addition, ASCs promoted the generation of splenic CD4+CD25+Foxp3+ Treg and Treg producing IL-10+ and TGF-β+ via an IDO dependent manner. Furthermore, the data revealed that IDO is upstream of Treg induction. The present study demonstrated that ASCs alleviate OB via IDO-induced Treg generation.

In mouse OB model, inflammation and infiltration of mononuclear cells were initiated quickly and peaked on day 7 after transplantation [29]. It has been hypothesized that the immunosuppressive effect of MSCs are induced by inflammatory cytokines and administration of MSCs at the peak of inflammation may enhance the beneficial outcomes [30]. Therefore, multiple doses of ASCs were selected to be administered on days 1, 3, 5, 8, 12, 15, 20 and 25 post-transplant. This may help to explain the discrepancy in another report that single dose of ASCs fails to alleviate the OB in a murine model [31]. Administration of MSCs with multiple doses has been proven to be safe. In a horse study, multiple injection of MSCs did not elicit a systemic inflammatory response and any other adverse effects [32]. Repeated intravenous infusion of MSCs improved cardiac function by attenuating myocardial collagen remodeling in a rat model of doxorubicin-induced dilated cardiomyopathy [33]. In a MSCs clinical trial for acute graft-versus-host disease, majority of patients received at least 2 doses of MSCs [34].

In the present study, while increased IDO expression was present in allografts, over-expression of IDO induced by ASCs infusion significantly alleviated OB. Similar findings have been reported in another study which over-expression of IDO via cDNA transfer resulted in prolonged corneal graft survival [25]. Several studies have recognized the function of IDO in transplantation tolerance. Over expression of IDO induces graft tolerance and attenuates acute rejection of tissue-engineered lung allografts in rats [35]. Prolongation of islet graft survival and spontaneous renal allograft acceptance may attribute to the function of IDO [36, 37]. In addition, kidney allograft tolerance induced by MSCs is associated with IDO expression [18]. Alterations in IDO may arise from elevated levels of IFN-γ. IFN-γ has long been documented as an inducer for IDO [38]. Furthermore, IFN-γ has been shown to trigger IDO activity in human monocyte-derived dendritic cells and endow allogeneic T cells with regulatory activity [39].

IDO may participate in graft tolerance via two mechanisms. First, elevated IDO activity results in depletion of tryptophan, an essential amino acid for T cell proliferation, which diminishes T cell responses. Second, IDO is able to facilitate the generation of Treg cells, which suppress a variety of physiological and pathological immune responses [40]. For example, IDO acts as a pivotal molecule controlling the functional status of Tregs following TLR9 ligation [41]. When MSCs are cocultured with peripheral blood mononuclear cells from patients of amyotrophic lateral sclerosis, IDO levels in the co-cultured MSCs are correlated with the increase in Treg induction [42]. In the present study, we showed that Treg levels were significantly increased in splenocytes after ASCs treatment. Furthermore, elevation of Treg was blocked by IDO inhibitor. However, Treg depletion did not affect IDO induction in the grafts. These results support the conclusion that ASCs mitigate OB via IDO-Treg mechanism.

MSCs have been shown to inhibit activation, proliferation, and function of immune cells, including T cells, B cells, NK cells, and dendritic cells [43]. MSCs are able to exert their effects via direct cell-to-cell contact and soluble factors produced by MSCs, such as nitric oxide, IDO, PGE2, and IL-10, in response to cytokines released by activated immune cells [44]. There is a species variation in the mechanisms of MSCs. Immunosuppression by cytokine-primed mouse MSCs is mediated by NO, whereas IDO is the main mediator for cytokine-primed human MSCs [30]. Human ASCs have been reported to modulate lymphocyte proliferation via IFN-γ–mediated IDO expression [45]. In the present study, our results revealed that the effects of human ASCs on mouse OB were blocked by an IDO inhibitor confirming the involvement of IDO-dependent mechanisms in human ASCs.

MSCs have been applied to treat graft rejection in transplant patients. In the first safety and feasibility study, infusion of autologous MSCs (1.7 to 2.0 million cells/kg) was conducted in two recipients of kidneys from living-related donors. There was a profound reduction in patient CD8+ T cell activity. There were also a progressive increase of the percentage of CD4+CD25+FoxP3+CD127− Treg and a marked inhibition of memory CD45RO+RA−CD8+ T cell expansion [46]. In a pilot study with 6 patients, kidney allograft recipients received two intravenous infusions (1 million cells/kg) of autologous BM-MSCs after showing signs of rejection and/or an increase in interstitial fibrosis/tubular atrophy. There was a resolution of tubulitis without interstitial fibrosis/tubular atrophy [47]. Another study evaluated the application of autologous MSCs as a replacement for IL-12 receptor antibody induction for 159 patients undergoing kidney transplantation. Patients were infused with BM-MSCs (0.8 − 2 million cells/kg) at kidney reperfusion and two weeks later. MSCs treatment resulted in lower incidence of acute rejection, decreased risk of opportunistic infection, and better estimated renal function at 1 year compared with IL-2 receptor antibody induction [48]. We can speculate that BM-MSCs and ASCs might also be beneficial to lung transplantation patients with BOS. Further studies are warranted to determine whether ASCs are a viable option for treating BOS in the future.

## Conclusions

ASCs engraft in allografts and elevate IDO expression in OB model. Infusion of multiple doses of ASCs reduce epithelial loss, epithelial apoptosis, trachea occlusion, and inflammatory response. ASCs decrease immune response via reducing CD3+ T-cell infiltration in the grafts and increase immune tolerance through inducing Treg generation in an IDO-dependent manner. These results indicate that ASCs might have the potential to become a novel therapeutic option to alleviate BOS after lung transplantation.

## Abbreviations

1-MT: 1-methyltryptophan
ASCs: Adipose-derived stem cells
BM: Bone marrow
BOS: Bronchiolitis obliterans syndrome
ELISA: Enzyme-linked immunosorbent assay
GAPDH: Glyceraldehyde-3-phosphate dehydrogenase
H&E: Hematoxylin and eosin
HTT: Heterotopic tracheal transplantation
IDO: Indoleamine 2, 3-dioxygenase
IFN-γ: Interferon-γ
IL-10: Interleukin-10
MSCs: Mesenchymal stem cells
NO: Nitric oxide
OB: Obliterative bronchiolitis
PBS: Phosphate-buffered saline
PGE2: Prostaglandin E2
TNF-α: Tumor necrosis factor-α
Treg: Regulatory T cell
TSG-6: Tumor necrosis factor-stimulated gene 6
TUNEL: Terminal deoxynucleotidyl transferase dUTP nick-end labeling
